# Corrective Intra-Articular Osteotomy Using a 3D-Printed Model and Induced Membrane Technique for AO/OTA C3 Distal Femur Fracture with Articular Malunion and Metaphyseal Nonunion

**DOI:** 10.1155/2020/1250231

**Published:** 2020-01-28

**Authors:** Natsumi Saka, Yoshinobu Watanabe, Gen Sasaki, Hirotaka Kawano

**Affiliations:** Department of Orthopaedics, Teikyo University School of Medicine, Japan

## Abstract

Comminuted distal femur fracture is a challenging injury, and care must be taken to reduce the articular fragment and acquire the sufficient stability for the metaphyseal comminution. We report the case of a AO/OTA C3-type distal femur fracture with articular malunion and metaphyseal nonunion. Articular malunion was treated with corrective osteotomy using a 3D-printed model for planning, and metaphyseal nonunion was treated with an induced membrane technique. *Conclusion*. Two major complications in the comminuted periarticular fracture can be addressed by an osteotomy and induced membrane technique. A 3D-printed model is a useful tool to evaluate the morphology of the malunited articular surface.

## 1. Introduction

A comminuted distal femur fracture poses challenges for surgical reconstruction. The intra-articular fracture should be reduced anatomically and fixed to prevent a decreased range of motion, pain, and osteoarthritis. Metaphyseal comminution, especially in open fractures is a well-documented risk of nonunion in literatures and internal fixation with sufficient stability is recommended [1]. Once that complication occurs, each complication has to be fixed to regain the lower limb function; however, approaching two issues is demanding. We have used two techniques for AO/OTA classification C3 (C3) distal femur fracture with articular malunion and metaphyseal nonunion. The corrective osteotomy was performed for articular malunion using a preoperative 3D-printed model. The large bone defect in metaphyseal nonunion was reconstructed using an induced membrane technique. Two significant complications of distal femur fracture can be solved using those techniques simultaneously. Of note, the 3D-printed model is a quite useful technique for planning corrective articular osteotomy of complex articular malunion.

For the malunion of fractures, salvage corrective osteotomy for extra-articular malunion is often reported, and intra-articular osteotomy for malunited Hoffa fracture (AO/OTA classification 33-B3) is also described [2–4]. However, there is no report of osteotomy in multiple segmental malunion of the distal femoral articular fracture.

For the nonunion with a large bone defect, the Ilizarov bone transfer transport technique or vascularized fibula autograft has been a traditional choice. However, there is a growing number of reports with an induced membrane technique for this type of large bone defect produced by trauma, infection, and tumor [5]. To our knowledge, there is no report of the articular malunion of C3 type treated with collective corrective osteotomy, combined with an induced membrane technique for metaphyseal nonunion. Herein, we report the salvage osteotomy for the malunited articular fragments of the C3-type distal femur fracture using a preoperative 3D-printed model and subsequent induced membrane technique for metaphyseal nonunion.

## 2. Case Presentation

**Figure 1:**
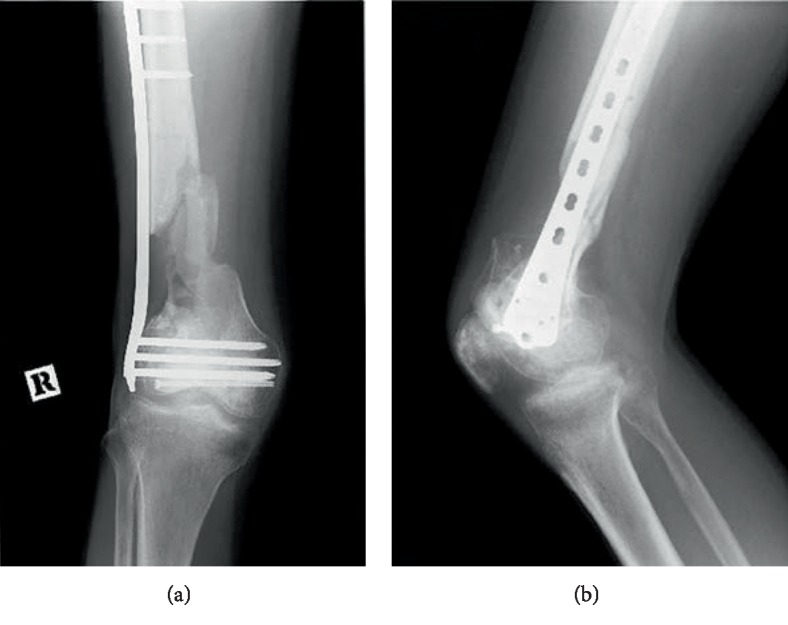
Preoperative radiograph. Anteroposterior (a) and lateral (b) radiograph of the right knee shows the metaphyseal nonunion of the femur.

**Figure 2:**
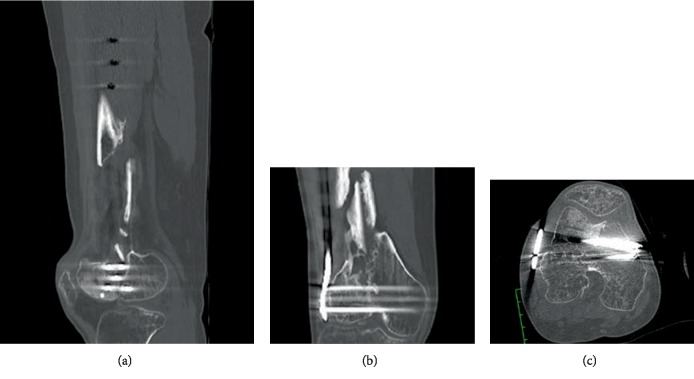
Preoperative CT scan of the right knee. Sagittal (a), coronal (b), and axial (c) CT scan shows the step-off between articular fragments.

A 30-year-old male has sustained an open distal femoral fracture (AO/OTA classification 33-C3, Gustilo-Anderson classification IIIA) from a motorcycle accident. He was initially treated with debridement, followed by open reduction and internal fixation with lateral locking plate two days after the injury. He was referred to our institution for the treatment of metaphyseal nonunion six months after the injury (Figure 1). Computed tomography (CT) also revealed malunited articular fragment with step-off (Figure 2). His femorotibial angle was 167°. At presentation, he could not bear weight on his injured leg, and the range of motion was -20° of knee extension and 90° of knee flexion. He underwent simultaneous corrective osteotomy for articular malunion and induced membrane technique for metaphyseal nonunion 14 months after the initial surgery.

### 2.1. Surgical Technique

**Figure 3:**
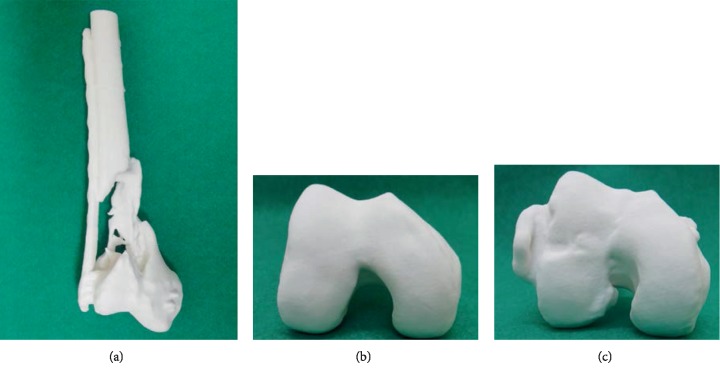
Preoperative 3D-printed model. A 3D-printed model was useful for visualizing metaphyseal nonunion (a) and articular malunion compared to the mirror image of the unaffected knee (b, c).

**Figure 4:**
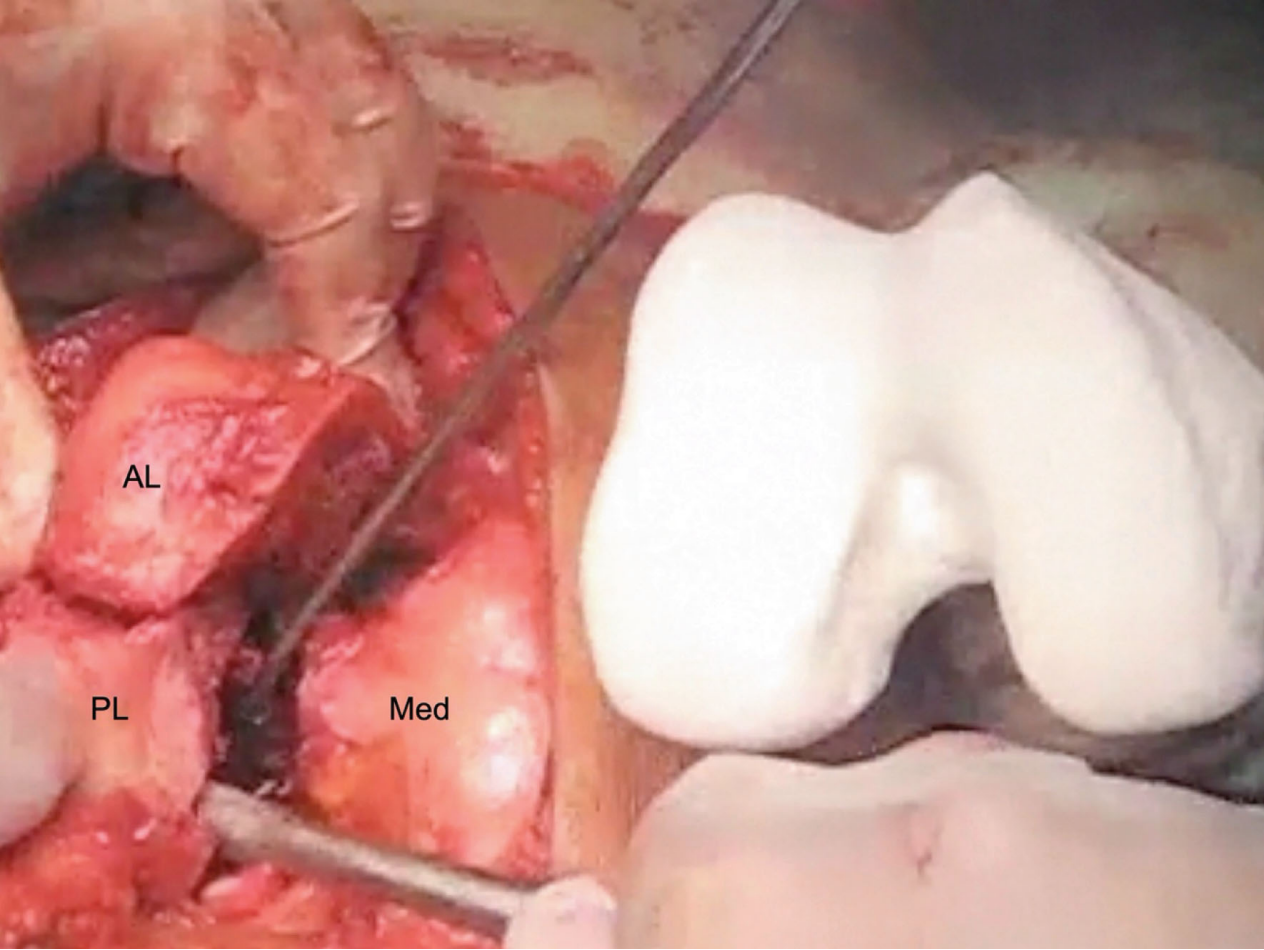
Intraoperative findings after the osteotomy of the articular fragment. Osteotomy was performed using a 3D-printed model for the comparison. AL: anterolateral fragment; PL: posterolateral fragment; Med: medial fragment.

**Figure 5:**
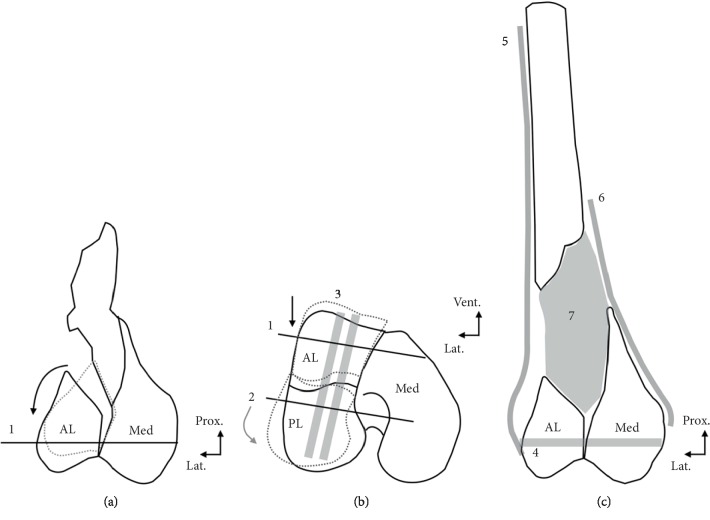
Illustrated figures of operative procedures. (a) Shows the distal femur seen from the coronal view. Malunited anterolateral fragment was described by a gray dotted line. Osteotomy of the malunited fragments was performed, and anterolateral fragment was temporally fixed to the medial fragment (1). Black arrow indicates the direction of the translation of the fragment at the osteotomy. (b) Shows the distal femur at the axial view. Malunited anterolateral and posterolateral fragments were described by a gray dotted line. Black arrow indicates the direction of the translation of the anterolateral fragment at the osteotomy. Gray arrow indicates the direction of the translation of the posterolateral fragment at the osteotomy. The posterolateral fragment was temporally fixed to the medial fragment (2). Anterolateral and posterolateral fragments were fixed with 6.5 mm cannulated screws (3). (c) Shows the distal femur seen from the coronal view. The anterolateral and medial fragments were fixed with a 6.5 mm cannulated screw (4). Lateral plate and medial plate were used to bridge the metaphyseal lesion and articular lesion (5, 6). Necrotic bone at the metaphyseal part was removed, and bone cement was filled to the bone defect (7). Prox: proximal; Lat: lateral; Vent: ventral; AL: anterolateral fragment; PL: posterolateral fragment; Med: medial fragment.

**Figure 6:**
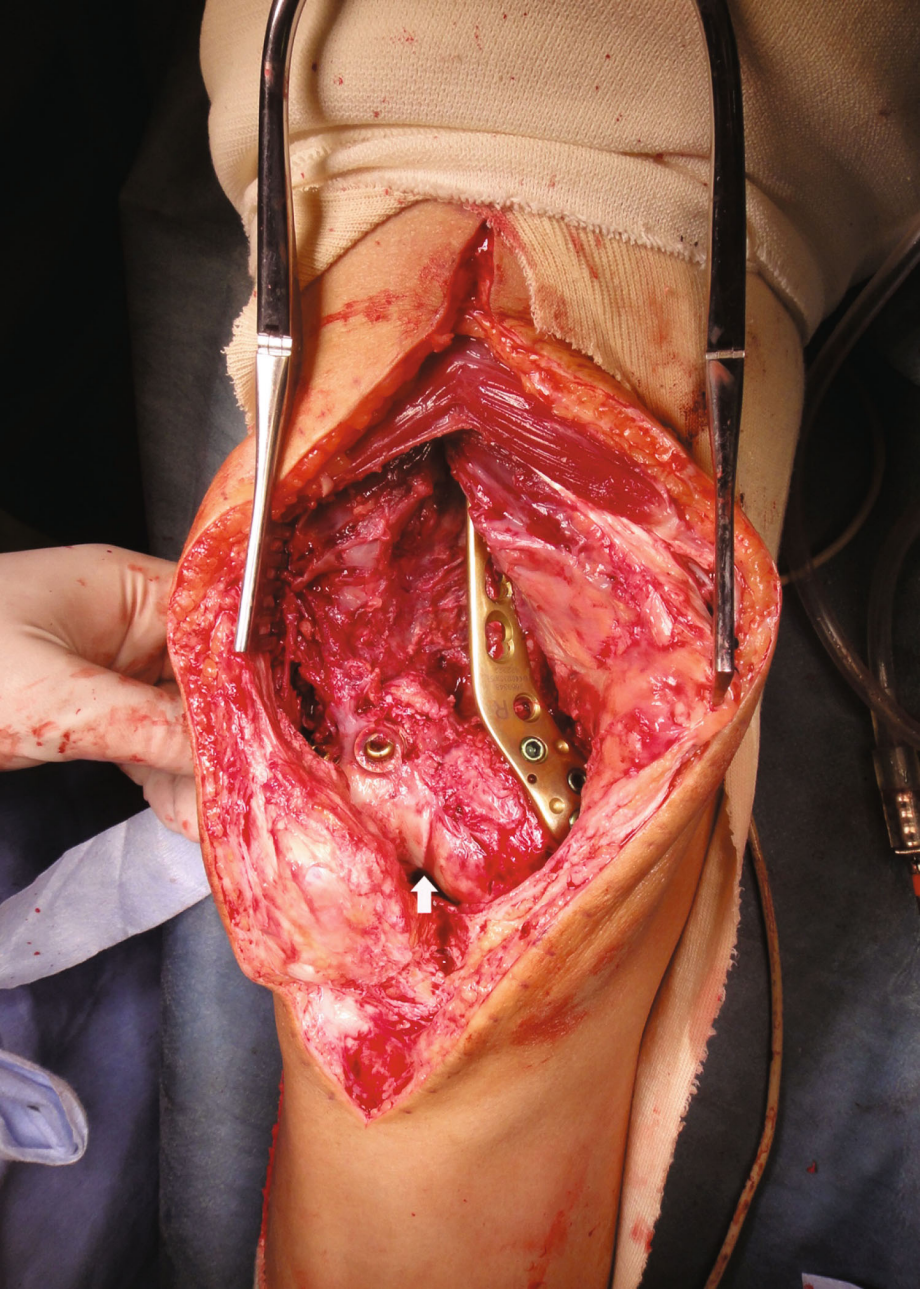
Intraoperative finding of the knee. It shows the fixed intercondylar step-off (white arrow) and defect of the metaphyseal area.

**Figure 7:**
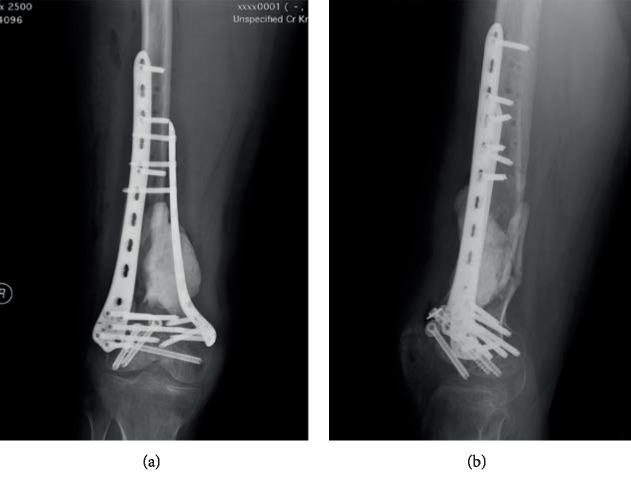
Postoperative radiograph of the right knee. Anteroposterior (a) and lateral (b) radiograph of the right knee shows the revision of internal fixation and cement placement in the bone defect.

**Figure 8:**
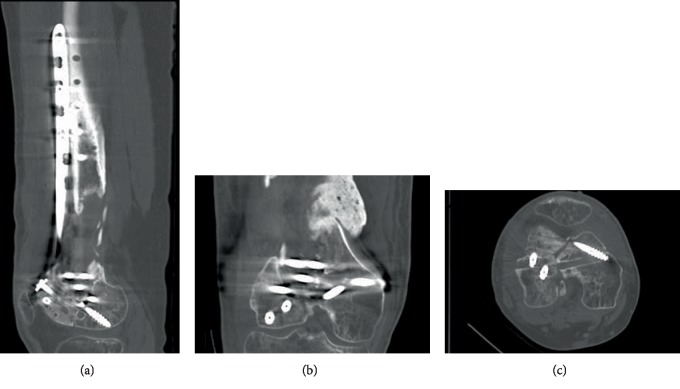
Postoperative CT scan of the right knee. Sagittal (a), coronal (b), and axial (c) CT scan of the knee shows the correction of articular step-off.

A preoperative 3D-printed model was made to gain the clearer picture of both articular malunion and metaphyseal nonunion (Figure 3). Malunited articular lesions were separated into three components. Longitudinal skin incision over the midline of the patella was placed. Subsequently, the medial parapatellar approach with the split in the quadriceps was used to acquire better exposure of the articular surface. Screws and the plate were removed with the additional incision over the iliotibial tract. Malunited articular fragments were separated into anterolateral, posterolateral, and medial fragments. Osteotomy of the malunited articular fragments was performed using an oscillating saw and chisel (Figure 4). The anterolateral fragment was moved distally and temporally fixed to the medial condyle fragment (Figure 5(a)). External rotation of the posterolateral fragment was corrected, and the fragment was temporally fixed to the medial condyle fragment (Figure 5(b)). The anterolateral and posterolateral fragments were fixed with two partially threaded 6.5 mm cannulated screws (Figure 5(b)). Subsequently, anterolateral and medial condyle fragments were fixed with one partially threaded 6.5 mm cannulated screw (Figure 5(c)). For the metaphyseal nonunion, necrotic bone fragments were removed, and the edge of each fragment was refreshed until the good hemorrhage from the bone marrow was confirmed, leaving a maximum of 9 cm defect. 4.5 mm LCP® Distal Femur Plate 9 Holes (DePuy Synthes®, USA) was placed over the lateral side of the femur. 3.5 mm LCP® Proximal Tibia (DePuy Synthes®, USA) was added in an inverted manner as a medial plate to provide more stability (Figures 5(c) and 6). Metaphyseal bone defect was filled with bone cement (Cemex fast Tecres® S.p.A, Italy) with vancomycin (Figure 5(c)). The wound was irrigated, and each layer was closed. Postoperative radiograph and CT scan showed the correction of valgus knee deformity and articular step-off (Figures 7 and 8). Six weeks after the osteotomy, bone grafting was performed. The bone cement in the metaphyseal part was removed, and the defect was filled with cancerous bone graft from the iliac crest. Both the articular osteotomy site and the metaphyseal nonunion demonstrated radiographic union at two months. For the remaining leg discrepancy, the patient underwent the additional distraction osteogenesis at the proximal femur with external fixator six months after the osteotomy.

**Figure 9:**
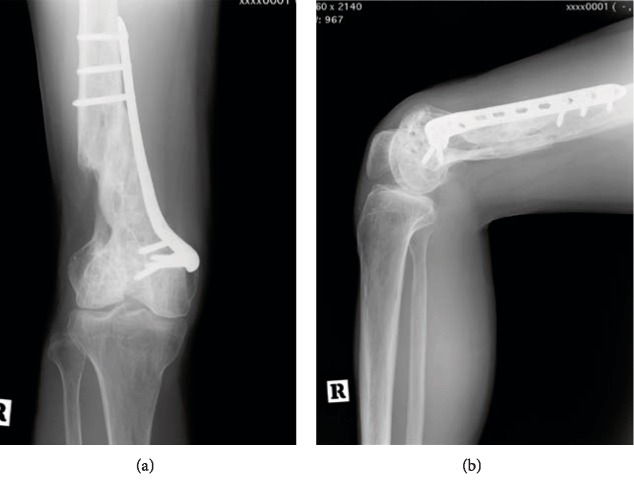
Radiograph of the knee at 2-year follow-up. Anteroposterior (a) and lateral (b) radiograph shows the union of the metaphyseal nonunion.

Six years after the osteotomy, the patient regained full weight bearing without pain (Figure 9). The range of motion has improved to 0° of knee extension and 120° of knee flexion, and the femorotibial angle is 172°. There is a mild bony spur formation over the tibia side of the knee, but there is no sign of necrosis of the epicondyle. The patient was informed that data from his case would be submitted for publication, and he provided consent.

## 3. Discussion

There are two unreported aspects regarding this case. Corrective osteotomy is useful for multiple articular nonunion in a C3-type distal femur fracture, and the preoperative 3D-printed model helped in understanding the morphological change of the malunited articular fragments. It can be used with the induced membrane technique for the coexisting metaphyseal nonunion.

Corrective osteotomy after a malunited intra-articular fracture is reported in other parts such as the distal radius and tibial plateau, which led to satisfactory results [6, 7]. There are two case reports of corrective osteotomy for the malunited Hoffa fracture [3, 4]. They have concluded that the malunited fracture can lead to the restriction of the range of movement, as well as pain and subsequent osteoarthritis. However, intra-articular corrective osteotomy for the C3-type distal femoral fracture has not been reported before. Different from two previous reports, this case was illustrated with both coronal and sagittal plane articular fracture malunion, which have led to the valgus deformity of the lower extremity as well as the step-off on the articular surface. Reducing the malunited articular fragment is important not only for regaining the articular surface but also for correcting the lower limb alignment.

One of the reasons why osteotomy for malunited complex articular fracture has not been performed might be the difficulty in the evaluation of each fragment. The 3D-printed model has become a matter of increasing interest, and its use is reported in the fixation of acute fractures as well as corrective osteotomy for the malunited femur diaphyseal fractures and malunited tibial plateau fractures [2, 8, 9]. For both extra-articular and intra-articular malunion, a 3D-printed model is reported as a useful way to reduce the risk of postoperative deformity and shorten the operation time [2, 9]. In our case, the preoperative 3D-printed model had helped us evaluate the gross deformity caused by malunion and nonunion.

The predominant risk for distal femur nonunion is metaphyseal comminution and open fracture, such as our case [1, 10]. Moreover, the reported nonunion rate after the lateral locking plate fixation is reported as high as 21% [10]. Osteotomy without jeopardizing vascularity and soft tissue coverage around the bone is of paramount importance for the success in the treatment of nonunion and malunion simultaneously. As a treatment for the nonunion of the femur, additional medial locking plate with simultaneous bone grafting can be chosen, but it has a limitation in the size of bone defect [11]. Since there is a risk of resorption, autologous bone grafting alone is not recommended when the defect exceeds 5 cm [12]. For the large bone defect, bone transport with external fixation or vascularized fibula autograft with the use of an external fixator has been a standard [13]. However, external fixation is complicated with soft tissue irritation, knee stiffness, and pin site infection. Besides, microsurgical skill is needed for vascularized fibula autograft. Masquelet et al. first introduced the induced membrane technique in 1986 as a solution for large bone defect produced by infection, trauma, and tumor [5, 14]. In this technique, bone defect is initially filled with bone cement and replaced by cancellous bone autograft in 6-8 weeks, after the surrounding membrane has produced. The cement spacer played roles as both mechanical stabilizer and inducer of the surrounding membrane which will provide the vascularization of the bone graft and prevent its resorption. This technique is advantageous in not using an external fixator nor microsurgical skill. In these contexts, there is a rapid rise of numbers in its use, including distal femur nonunion [15]. In our case, the maximum bone defect size was 9 cm, and the induced membrane technique would be a matter of choice, rather than one-stage osteotomy and bone grafting.

As a conclusion, corrective osteotomy for the C3-type distal femur fracture with an induced membrane technique is a useful method for both articular malunion and metaphyseal nonunion. Such a technique can be used in a fracture in other joints which involves both articular nonunion and metaphyseal nonunion. The 3D-printed model is a useful method for the planning and evaluation of the osteotomy.
